# Distinct parafacial regions in control of breathing in adult rats

**DOI:** 10.1371/journal.pone.0201485

**Published:** 2018-08-10

**Authors:** Robert T. R. Huckstepp, Kathryn P. Cardoza, Lauren E. Henderson, Jack L. Feldman

**Affiliations:** Department of Neurobiology, David Geffen School of Medicine, University of California Los Angeles, Los Angeles, California, United States of America; National Yang-Ming University, TAIWAN

## Abstract

Recently, based on functional differences, we subdivided neurons juxtaposed to the facial nucleus into two distinct populations, the parafacial ventral and lateral regions, i.e., pF_V_ and pF_L_. Little is known about the composition of these regions, i.e., are they homogenous or heterogeneous populations? Here, we manipulated their excitability in spontaneously breathing vagotomized urethane anesthetized adult rats to further characterize their role in breathing. In the pF_L_, disinhibition or excitation decreased breathing frequency (*f*) with a concomitant increase of tidal volume (V_T_), and induced active expiration; in contrast, reducing excitation had no effect. This result is congruent with pF_L_ neurons constituting a conditional expiratory oscillator comprised of a functionally homogeneous set of excitatory neurons that are tonically suppressed at rest. In the pF_V_, disinhibition increased *f* with a presumptive reflexive decrease in V_T_; excitation increased *f*, V_T_ and sigh rate; reducing excitation decreased V_T_ with a presumptive reflexive increase in *f*. Therefore, the pF_V_, has multiple functional roles that require further parcellation. Interestingly, while hyperpolarization of the pF_V_ reduces ongoing expiratory activity, no perturbation of pF_V_ excitability induced active expiration. Thus, while the pF_V_ can affect ongoing expiratory activity, presumably generated by the pF_L_, it does not appear capable of directly inducing active expiration. We conclude that the pF_L_ contains neurons that can initiate, modulate, and sustain active expiration, whereas the pF_V_ contains subpopulations of neurons that differentially affect various aspects of breathing pattern, including but not limited to modulation of ongoing expiratory activity.

## Introduction

Several brainstem motor nuclei are surrounded by respiratory-related neurons [1, 2]. In the case of the facial nucleus, parafacial neurons are essential components of the breathing central pattern generator (bCPG). In particular, parafacial neurons that express the neurokinin-1 receptor (NK1R), the homeobox gene Phox2b, and the glutamate transporter VGlut2, are essential to CO_2_ chemoreception [3–6]; notably, a subpopulation of these neurons have rhythmic respiratory-related activity, both *in vitro* and *in vivo* [7–9], leading us to postulate that breathing is driven by a dual oscillator system [10]. We identified two neighboring parafacial regions, lateral (pF_L_) and ventral (pF_V_) that appear to be functionally distinct components of the bCPG [11]. We hypothesized that the pF_L_ is a conditional expiratory oscillator that is inhibited at rest [8, 11, 12], whereas the pF_V_ provides a generic source of excitatory drive for both inspiration and expiration whose activity depends, at least in part, on CO_2_-related signals [11, 13–15]. Furthermore, two parafacial subpopulations, containing Gastrin-Releasing Peptide and Neuromedin B (GRP and NMB, respectively) modulate sighing [16]. Therefore, further subdivision of the parafacial region into functionally distinct nuclei may be warranted, as is the case for other subcortical brain regions, such as the nucleus tractus solitarius, periaqueductal gray, and paraventricular nucleus [17–19]. To further investigate the functional contributions of the pF_L_ and pF_V_, we selectively modulated their excitability and measured the effects on ventilation in spontaneously breathing vagotomized urethane anesthetized adult rats.

We conclude that the: i) pF_L_ contains a functionally homogenous population of excitatory neurons that are tonically inhibited at rest, which following an increase in excitability can initiate and maintain active expiration; ii) pF_V_ contains at least four functionally distinct subpopulations of neurons: three subpopulations that are tonically inhibited at rest, which can separately affect *f*, modulate active expiration, and modulate basal sigh rate, and one tonically active subpopulation that predominately affects V_T_. Interestingly no subpopulation of pF_V_ neurons appears capable of directly inducing active expiration; instead the pF_V_ modulates active expiration generated elsewhere, presumably by effects in the pF_L_ and/or (pre)motoneuron pools.

## Methods

All protocols were approved by the University of California Los Angeles Chancellor’s Animal Research Committee. All experiments were performed in spontaneously breathing vagotomized urethane anesthetized adult Male Sprague-Dawley rats (350–450 g) rats.

### Ventral approach

**Fig 1:**
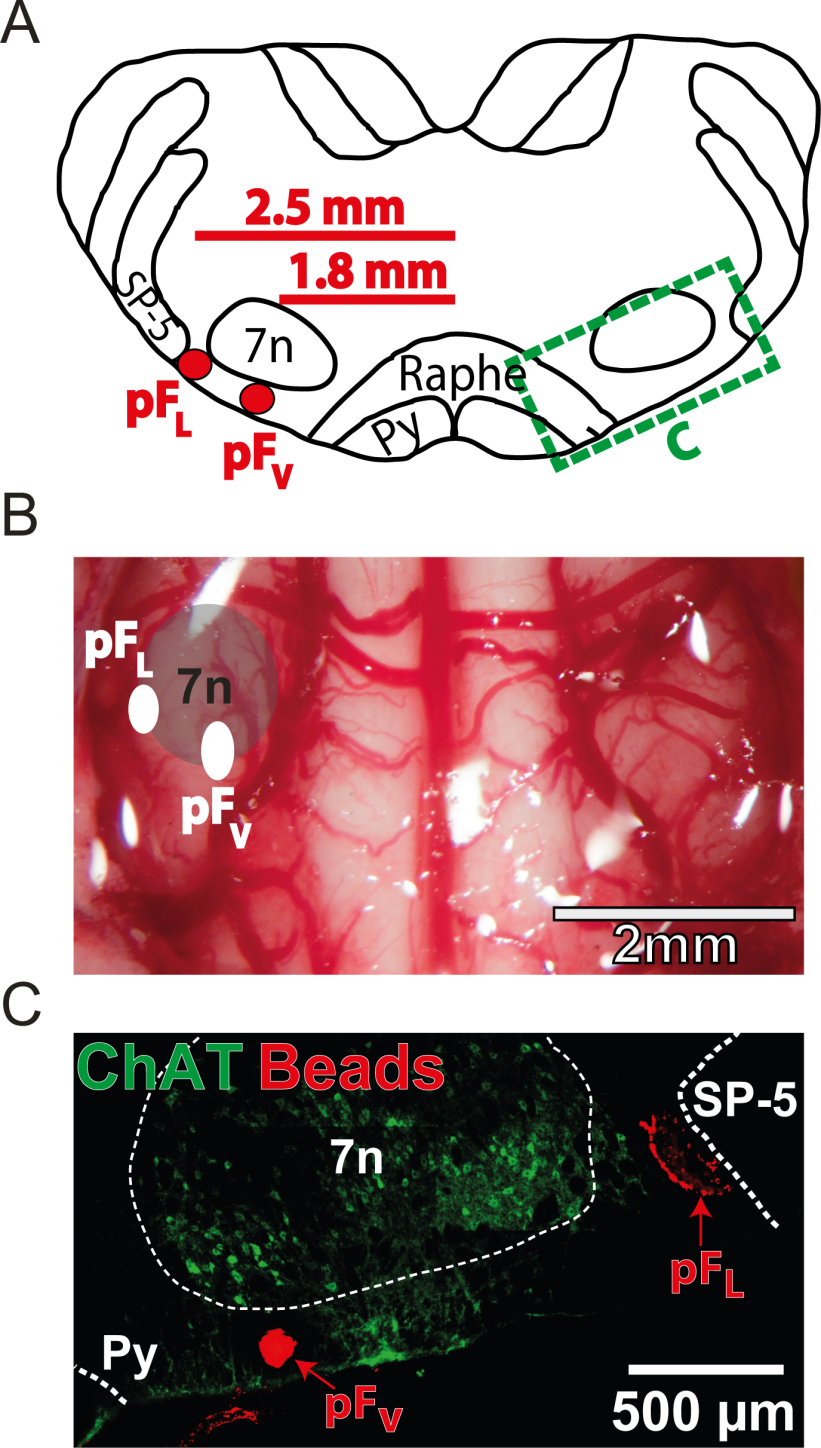
Histological analysis of parafacial regions. A) Localization of injections into pF_V_ and pF_L_. Transverse view of medulla at Bregma -11.25 mm. Red circles show locations of injection sites for pF_V_ and pF_L_. Green dashed box is magnified in C. B) Ventral view of medullary surface with location of pF_V_ and pF_L_ injection sites, marked by white circles, superimposed. C) Micrographs of injection sites. Green marks staining for choline acetyl transferase (ChAT), highlighting the cholinergic neurons of the facial (VII) nucleus, and red marks fluorescent beads coinjected with micropipette solutions in to the pF_V_ and pF_L_. Py–Pyramidal tract, SP-5 –Spinal trigeminal tract, 7n –Facial nucleus.

Anesthesia was induced with isofluorane and maintained with urethane (1.2–1.7 g/kg; Sigma) in sterile saline via a femoral catheter. Rats were placed supine in a stereotaxic apparatus on a heating pad to maintain body temperature at 37±0.5°C. The trachea was cannulated. Respiratory flow was monitored via a flow head (GM Instruments), and CO_2_ via a capnograph (Type 340: Harvard Apparatus) connected to the tracheal tube. Paired electromyographic (EMG) wires (Cooner Wire Co.) were inserted into genioglossal (GG), diaphragmatic (Dia), and oblique abdominal muscles (Abd). Anterior neck muscles were removed, a basiooccipital craniotomy exposed the ventral medullary surface, and the dura was resected. After bilateral vagotomy, exposed tissue around the neck and mylohyoid muscle were covered with dental putty (Reprosil; Dentsply Caulk) to prevent drying. Rats were left for 30 minutes for breathing to stabilize. At rest, ventilation consisted of alternating active inspiration and passive expiration. Once stabilized, solutions of drugs in micropipettes were pressure injected (100–200 nL) bilaterally using a Picospritzer II (General Valve Corp.) controlled by a Master 8 pulse generator (AMPI) into the pF_L_ or pF_V_ (Fig 1). To reduce disruption of the tissue, solutions were injected at ~50 nL/min. To ensure parity of injections of different drugs, i.e., AMPA, B+S, A+N, and consistency between both sites, i.e., pF_L_ and pF_V_, the bilateral injections of a drug were performed ~2 mins apart. The timing between; the 2 injections of AMPA (119 ± 16 sec), the 2 injections of B+S (121 ± 10 secs), and the 2 injections of A+N (121 ± 15 secs) were not statistically different (F_[2, 47]_ = 0.01; p = 0.98; 2-way ANOVA), and no differences were found between the timings of the 2 injections in the pF_V_ (121 ± 8 secs) and the 2 injections in the pF_L_ (120 ± 13 secs; F_[1,47]_ = 0.0004; p = 0.98; 2-way ANOVA). The timing between the 2 injections of Glu before (122 ± 13 secs) and after (120 ± 13 secs) vagotomy, were also not statistically different (p = 0.8; paired T-test). After each injection rats were allowed 30–45 minutes for drugs to take effect and washout, and for baseline recordings to stabilize before the next injection.

The pF_L_ is defined as the area ventral to the lateral edge of the facial nucleus, juxtaposed to the spinal trigeminal tract [11]. The pF_V_ is defined as the area ventral to the caudal half of the facial nucleus, at a central location between the pyramidal tract and the spinal trigeminal tract [11]. Coordinates: lateral from the basilar artery, rostral from the rostral hypoglossal nerve rootlet, and dorsal from the ventral surface (in mm); pF_V_: 1.8, 0.6, 0.1, and pF_L_: 2.5, 0.9, 0.2.

Injections contained: i) bicuculline methylbromide (250 μM; Tocris) and strychnine hydrochloride (250 μM; Sigma) (B+S) to antagonize GABA_A_ and glycine receptors, respectively. Injections of B+S led to disinhibition of the pF_L_ (B+S_pFL_) or pF_V_ (B+S_pFV_); ii) AMPA (20 μM; Sigma) to activate glutamatergic AMPA receptors. Injections of AMPA lead to excitation of the pF_L_ (AMPA_pFL_) or pF_V_ (AMPA_pFV_) or; iii) 2-amino-5-phosphopentanoic acid (AP-5; 1mM; Sigma) and 2,3-dihydroxy-6-nitro-7-sulfamoyl-benzo[f]quinoxaline-2,3-dione (NBQX; 1mM; Sigma) (A+N) to antagonize glutamatergic NMDA and AMPA receptors, respectively. Injections of A+N reduced excitation in the pF_L_ (A+N_pFL_) or pF_V_ (A+N_pFV_). All drugs were diluted in sterile saline balanced with NaOH to pH 7.35.

In one set of experiments, a ventral approach to the medulla was performed in vagus-intact rats. After a resting period to allow breathing to stabilize, rats received 100–200 nL bilateral injections of glutamate (10 mM; Sigma) administered at ~50 nL/min into the pF_V_ (Glu_pFV_), following which breathing was allowed to recover. After breathing returned to baseline levels, rats were bilaterally vagotomized at the mid-cervical level. Breathing was allowed to stabilize (~30–60 mins), following which rats received a second bilateral injection of Glu_pFV_.

Care was taken to reduce any transient effects of mechanical stimulation when placing the pipette into the tissue. As experimental controls to determine whether insertion of the pipette and injection of solution *per se* had effects, we tested the effects of saline injections.

All injections contained fluorescent beads (red fluoSpheres; Invitrogen) to allow for post-hoc identification of injection sites.

### Localization of injection sites (Fig 1)

Rats were sacrificed by overdose of urethane and transcardially perfused with saline followed by cold (4°C) paraformaldehyde (PFA; 4%). The medulla was harvested and postfixed in 4% PFA overnight at 4°C, then cryoprotected in sucrose (30%) in standard PBS (1–3 days at 4°C). PBS contained (mM): NaCl 137, KCl 2.7, Na_2_HPO_4_ 10, KH_2_PO_4_ 1.8, pH 7.4. Brainstems were transversely sectioned at 40 μm. Free-floating sections were incubated overnight in PBS containing 0.1% Triton X-100 (PBT) and mouse anti-NeuN primary antibody (1:500; EMD Millipore) or goat anti-cholineacetyl transferase (ChAT; 1:100; EMD Millipore). The tissue was washed in PBS, 6 times for 5 minutes per wash, and then incubated separately for 2–4 hours in a solution of PBT containing either donkey anti-mouse Alexa Fluor 647 secondary antibody (1:250; Jackson ImmunoResearch Laboratories, Inc.) or donkey anti-goat Alexa Fluor 488 (1:250; Invitrogen), for NeuN and ChAT, respectively. The tissue was washed in PBS, 6 times for 5 minutes. Slices were mounted onto polylysine-coated slides, dehydrated overnight at 22°C, and coverslipped using Cytoseal 60 (Electron Microscopy Sciences). Slides were analyzed using a fluorescent microscope with AxioVision acquisition software (AxioCam2, Zeiss).

### Data analysis and statistics

EMG signals and airflow measurements were collected using preamplifiers (P5; Grass Instruments) connected to a Powerlab AD board (ADInstruments) in a computer running LabChart software (ADInstruments), and were sampled at 400 Hz/channel. High pass filtered (>0.1 Hz) flow head measurements were used to calculate: tidal volume (V_T_, peak amplitude of the integrated airflow signal during inspiration; pressure sensors were calibrated with a 3 mL syringe); V_T_ is expressed as mL. Inspiratory duration (T_I_, beginning of inspiration until peak V_T_), expiratory duration (T_E_, peak V_T_ to the beginning of the next inspiration), and *f* (1/[T_I_+T_E_]); T_I_ and T_E_, are expressed in secs (s), and *f* is expressed as breaths per minute (BPM). Minute ventilation (V_e_) was calculated as *f* x V_T_, and is expressed as mL/min. EMG data were integrated (τ = 0.05 s; ∫Dia_EMG_, ∫GG_EMG_, and ∫Abd_EMG_ in arbitrary units (a.u.)) and the peak amplitude of each signal computed for each cycle.

To obtain control values, all parameters were averaged for 20 respiratory cycles preceding each injection. To measure drug effect, 20 cycles were averaged during a period where the injection had its greatest effect on the airflow channel. Measurements were only made of the initial response to the drug, usually within the first 5 minutes following the 2^nd^ injection, at a similar time as the expanded traces in the figures (marked in each figure by a black arrow with a black dotted line). Care was taken to avoid measurements where reflexive changes had taken place, for example, where the drug caused an initial decrease in breathing followed by a compensatory increase in breathing as the compound wore off. In these cases, measurements were taken at the peak effect during initial decrease and not during the reflexive increase that followed. Data was analyzed offline and exported to Excel™ (Microsoft) for further analysis. All statistical tests were performed using Igor Pro™ (WaveMetrics), except 2-way ANOVAs which were performed in OriginPro™ (OriginLab).

As described above, for each rat we calculated the average of 20 cycles preceding the stimulus (_control_), and the average of 20 cycles during the stimulus (_stimulus_). Both groups, the _control_ values and their associated _stimulus_ value for every rat, were combined into a single data set. To facilitate graphical comparisons data was normalized to the highest value in the data set regardless of whether it belonged to _control_ or _stimulus_ group. Therefore, the highest value in the data set, whether it be _control_ or _stimulus_, was 1.0.

We define active expiration as the epoch of appearance of burst activity in expiratory muscles, i.e., abdominals, that leads to forced air outflow, typically during late expiration, and consequently, increased V_T_ in the following inspiration. We define sighs by their characteristic augmented V_T_ caused by a second inspiratory effort that occurs before the initial eupneic inspiration is complete. These augmented breaths result from largely from high amplitude inspiratory Dia_EMG_.

Data were not normally distributed. Data were therefore analyzed using the non-parametric 2-sided Wilcoxon signed-rank test with a significance level of P ≤ 0.05 and reported as median and interquartile range (IQR). Data are displayed as box and whisker plots for comparison of groups, and as line graphs for individual experiments. There were 8 biological repeats and no technical repeats in all data sets, with 2 exceptions: Statistical outliers were excluded from the data if they failed both Pierce’s criterion and Grubb’s test; this led to the removal of 1 outlier from the Abd_EMG_ data from the AMPA_pFV_ and AMPA_pFL_ data sets.

Power analysis was calculated in G*Power3 (http://www.gpower.hhu.de/en.html [20]), using a Wilcoxon signed rank tests (matched pairs): with an α error probability of 0.05, and a power (1-β error probability) of 0.90, and effect sizes were calculated from the data (Table 1).

**Table 1:** Statistical analysis.

PARAMETER	DATA STRUCTURE	TYPE OF TEST	EFFECT SIZE	POWER	REQUIRED SAMPLE SIZE
***f***	Non-parametric	2-sided Wilcoxon signed-rank test	1.5	0.94	n = 8
**T_I_**	Non-parametric	2-sided Wilcoxon signed-rank test	1.7	0.95	n = 7
**T_E_**	Non-parametric	2-sided Wilcoxon signed-rank test	1.8	0.92	n = 6
**V_T_**	Non-parametric	2-sided Wilcoxon signed-rank test	1.4	0.94	n = 8
**Dia_EMG_**	Non-parametric	2-sided Wilcoxon signed-rank test	1.4	0.94	n = 8
**GG_EMG_**	Non-parametric	2-sided Wilcoxon signed-rank test	1.7	0.95	n = 7
**Abd_EMG_**	Non-parametric	2-sided Wilcoxon signed-rank test	18.5	1.0	n = 3

## Results

### Disinhibition of pF_L_ or pF_V_ affect breathing pattern (Figs 2–5, Table 2)

**Fig 2:**
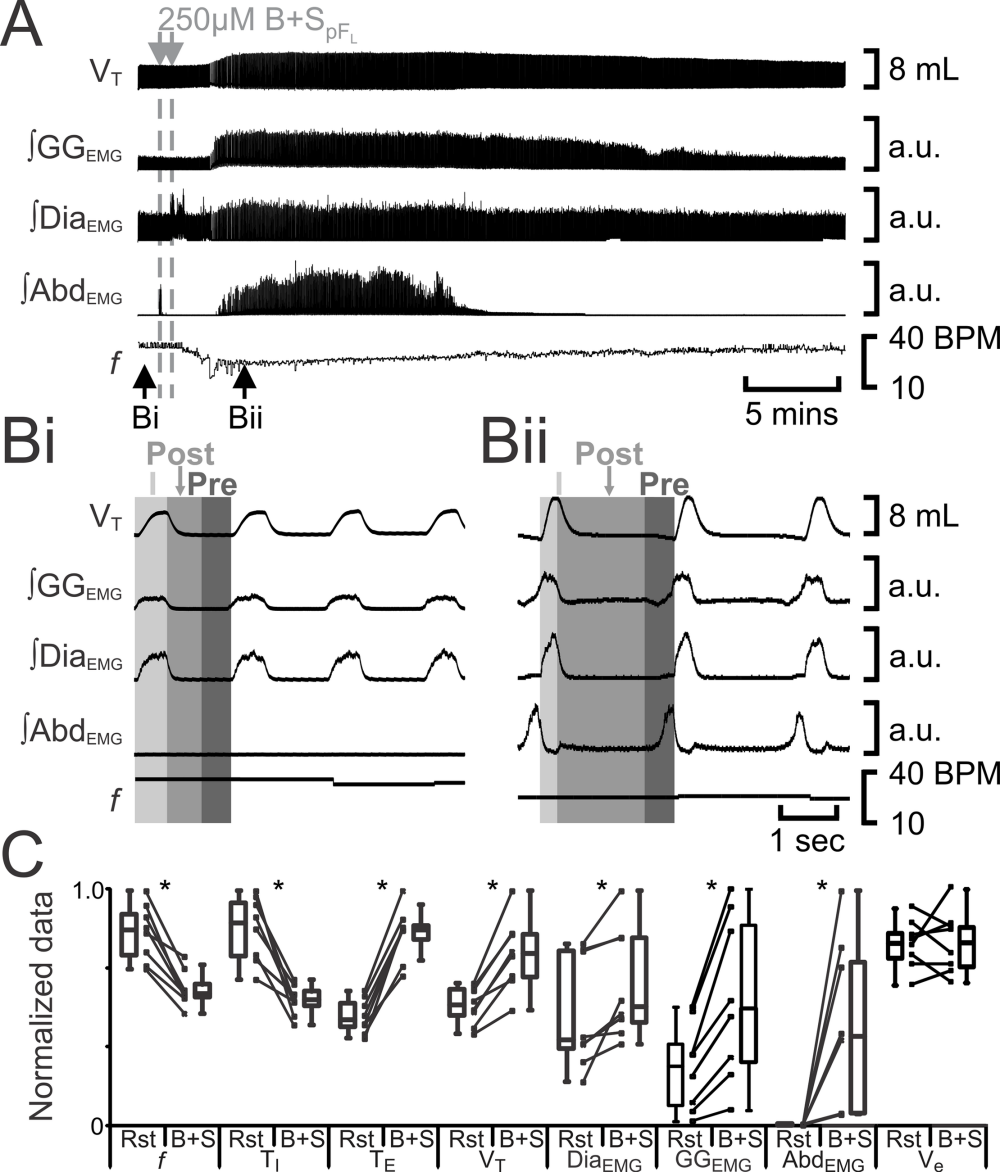
B+S_pFL_ induces active expiration. A) Integrated traces from a single experiment. Black arrows at bottom indicate epochs in expanded traces (Bi and Bii), gray arrows at top indicate unilateral injections for B+S_pFL_. Bi) Rest. Bii) Following B+S_pFL_. Grey vertical boxes demark period of each breath taken up by inspiration (I; light gray), post-inspiration (Post: medium grey), and pre-inspiration (Pre: Dark gray). C) Comparison between ventilation at rest (Rst) and after B+S_pFL_ injection. Lines connect data from individual experiments, box and whisker plots show combined data. Data are normalized to highest value for each parameter, i.e., *f*, T_I,_ T_E_, V_T_, GG_EMG_, Dia_EMG_, or Abd_EMG_ regardless of whether it belonged to control or B+S_pFL_ group. *: p < 0.05. frequency–*f*, T_I_−inspiratory period T_E_,–expiratory period, tidal volume–V_T_, GG_EMG_−genioglossus electromyogram, Dia_EMG_−diaphragm electromyogram, Abd_EMG_−abdominal electromyogram. BPM–breaths per minute, a.u.—arbitrary units.

Disinhibition of pF_L_ neurons by the GABAergic antagonist bicuculline and the glycine antagonist strychnine (B+S_pFL_) can induce active expiration [8, 11], which we confirm here. Bilateral injection of B+S_pFL_ (n = 8) decreased *f* and T_I_, increased T_E_, V_T_, ∫Dia_EMG_, and inspiratory-related ∫GG_EMG_ activity, and induced rhythmic expiratory bursting in ∫GG_EMG_ and ∫Abd_EMG_ (Fig 2), the latter a signature of active expiration, q.v., [8, 11]. Bilateral B+S_pFL_ had no effect on minute ventilation (V_e_) due to a compensatory increase in V_T_ in response to the changes in *f* elicited by the antagonism of the inhibitory receptors.

**Fig 3:**
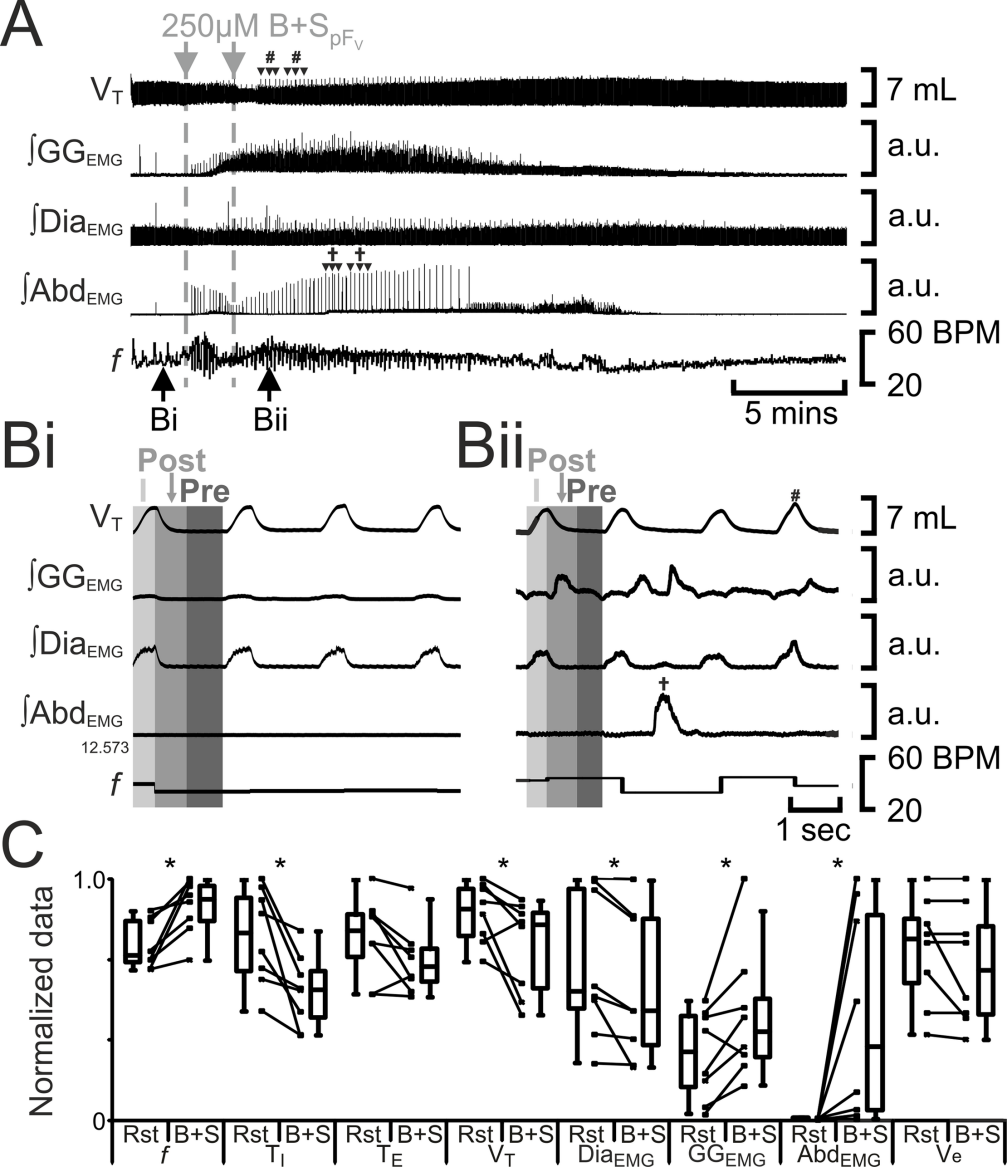
B+S_pFV_ increases *f*, decreases V_T_, and induces post-inspiratory activity in abdominal muscles and pre- and post-inspiratory activity in gengioglossus muscles. A) Integrated traces from a single experiment. Black arrows at bottom indicate epochs in expanded traces (Bi and Bii), gray arrows at top indicate unilateral injections for B+S_pFV_. Examples of sighs are marked with arrowheads labelled with #. Post-inspiratory burst Abd_EMG_s are marked with arrowheads labelled with †. Bi) Rest. Bii) Following B+S_pFV_. Grey vertical boxes demark phases of each breath: inspiration (I; light gray), post-inspiration (Post: medium grey), and pre-inspiration (Pre: Dark gray). Sigh marked by #. Post-Inspiratory Abd_EMG_ marked by †. C) Comparison between ventilation at rest (Rst) and after B+S_pFV_ injection. Lines connect data from individual experiments, box and whisker plots show combined data. Data are normalized to highest value for each parameter, i.e., *f*, T_I,_ T_E_, V_T_, GG_EMG_, Dia_EMG_, or Abd_EMG_ regardless of whether it belonged to control or B+S_pFL_ group. *: p < 0.05. Abbreviations defined in Fig 2.

**Fig 4:**
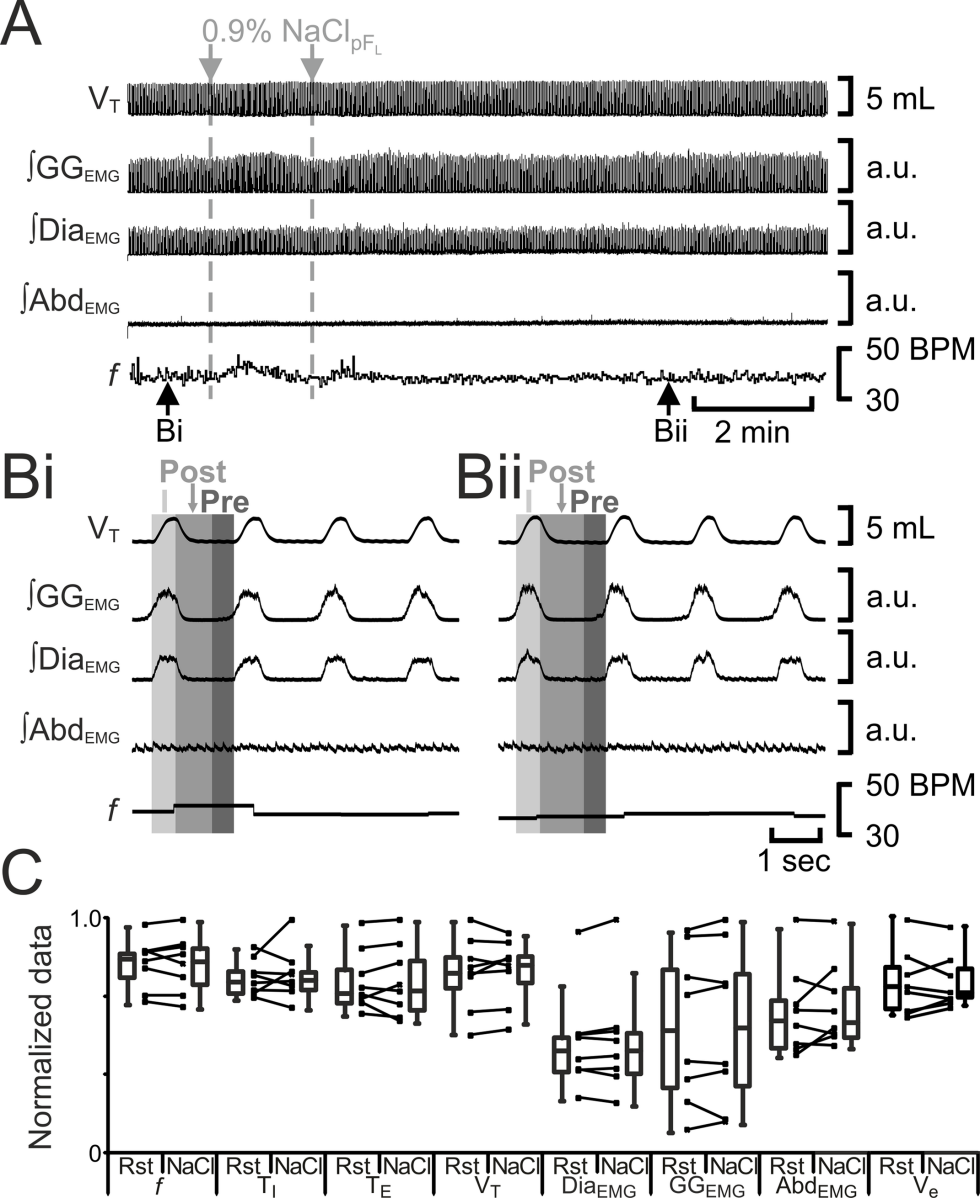
Saline_pFL_ does not affect breathing. A) Integrated traces from a single experiment. Black arrows at bottom indicate epochs in expanded traces (Bi and Bii), gray arrows at top indicate unilateral injections for Saline_pFL_. Bi) Rest. Bii) Following Saline_pFL_. Grey vertical boxes demark phases of each breath: inspiration (I; light gray), post-inspiration (Post: medium grey), and pre-inspiration (Pre: Dark gray). a.u.: arbitrary units. C) Comparison between ventilation at rest (Rst) and after Saline_pFL_. Lines connect data from individual experiments, box and whisker plots show combined data. Data are normalized to highest value for that parameter, i.e., *f*, T_I,_ T_E_, V_T_, GG_EMG_, Dia_EMG_, or Abd_EMG_ regardless of whether it belonged to control or saline_pFL_ group. Abbreviations defined in Fig 2.

**Fig 5:**
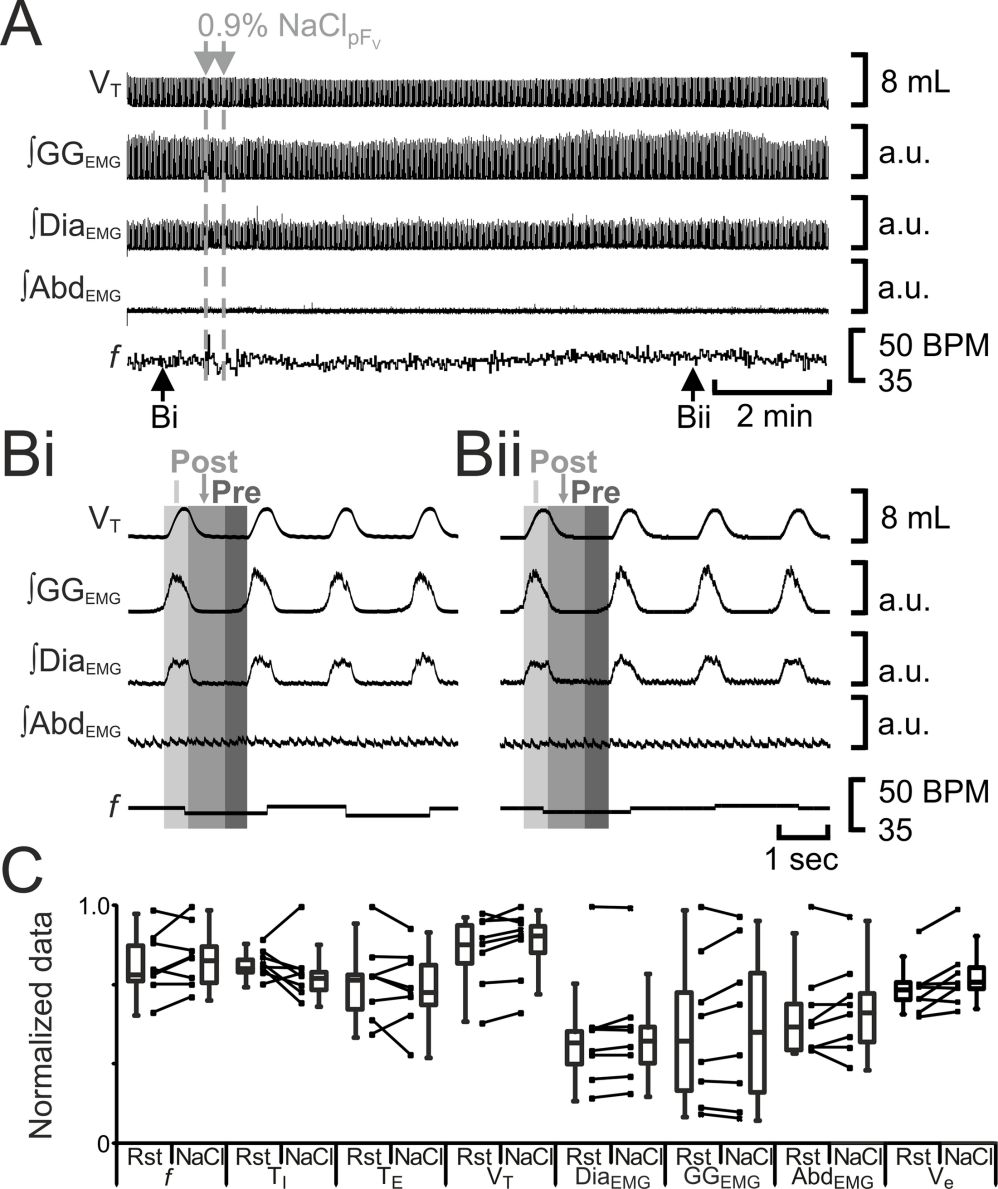
Saline_pFV_ does not affect breathing. A) Integrated traces from a single experiment. Black arrows at bottom indicate epochs in expanded traces (Bi and Bii), gray arrows at top indicate unilateral injections for Saline_pFV_. Bi) Rest. Bii) Following Saline_pFV_. Grey vertical boxes demark phases of each breath: inspiration (I; light gray), post-inspiration (Post: medium grey), and pre-inspiration (Pre: Dark gray). a.u.: arbitrary units. C) Comparison between ventilation at rest (Rst) and after Saline_pFV_. Lines connect data from individual experiments, box and whisker plots show combined data. Data are normalized to highest value for that parameter, i.e., *f*, T_I,_ T_E_, V_T_, GG_EMG_, Dia_EMG_, or Abd_EMG_ regardless of whether it belonged to control or Saline_pFV_ group. Abbreviations defined in Fig 2.

**Table 2:** Median and interquartile range for all recorded variables.

**A) *pF***_***L***_	***f*** **(BPM)**	**T**_**I**_ **(secs)**	**T**_**E**_ **(secs)**	**V**_**T**_ **(mL)**	**Dia**_**EMG**_ **(a.u.)**	**GG**_**EMG**_ **(a.u.)**	**Abd**_**EMG**_ **(a.u.)**	**V**_**e**_ **(mL·min**^**-1**^**)**
**Rest**	44, 9	0.5, 0.1	1.0, 0.2	5.3, 1.1	26, 30	9, 9	0, 0	218, 30
**B+S**	28, 3	0.3, 0.0	1.8, 0.1	7.5, 1.9	36, 26	17, 20	16, 27	219, 47
**P =**	0.008	0.008	0.008	0.008	0.008	0.008	0.008	0.9
**Rest**	45, 8	0.4, 0.1	0.9, 0.3	5.2, 1.1	25, 22	6, 8	0, 0	212, 52
**AMPA**	32, 14	0.3, 0.1	1.5, 0.8	6.1, 1.0	29, 23	13, 10	2, 3	177, 7
**P =**	0.008	0.02	0.008	0.008	0.02	0.008	0.02	0.1
**Rest**	54, 11	0.3, 0.1	0.7, 0.2	4.2, 0.7	28, 24	6, 8	0, 0	215, 43
**A+N**	59, 10	0.4, 0.1	0.6, 0.2	3.9, 1.1	27, 25	6, 7	0, 0	213, 28
**P =**	0.4	0.4	0.3	0.8	0.5	0.2	0.4	0.9
**B) *pF***_***V***_								
**Rest**	37, 9	0.5, 0.2	1.1, 0.9	5.4, 1.2	28, 26	11, 11	0, 0	207, 22
**B+S**	50, 8	0.3, 0.1	0.9, 0.2	5.0, 1.9	24, 28	14, 9	7, 18	229, 80
**P =**	0.008	0.008	0.054	0.02	0.008	0.04	0.008	0.5
**Rest**	42, 6	0.5, 0.1	1.0, 0.2	5.2, 0.7	25, 29	7, 77	0, 0	210, 39
**AMPA**	47, 4	0.3, 0.1	0.9, 0.2	5.5, 1.2	30, 28	13, 11	0, 0	262, 40
**P =**	0.02	0.008	0.2	0.04	0.02	0.008	0.7	0.02
**Rest**	48, 8	0.43, 0.1	0.8, 0.2	4.7, 0.9	28, 24	7, 8	0, 0	217, 16
**A+N**	61, 22	0.36, 0.1	0.6, 0.2	3.7, 0.8	26, 23	3, 5	0, 0	205, 33
**P =**	0.02	0.02	0.04	0.008	0.02	0.008	0.4	0.3
**C) *Glutamate-pF***_***V***_						
**VI Rest**	104, 53	0.21, 0.08	0.3, 0.1	2.1, 0.7	15, 13	3, 4	0, 0	254, 34
**VI Glu**	87, 39	0.23, 0.09	0.4, 0.2	2.4, 0.9	16, 15	5, 5	0, 0	232, 51
**P =**	0.03	0.02	0.02	0.046	0.02	0.02	0.9	0.4
**Vx Rest**	46, 20	0.34, 0.26	0.8, 0.4	2.8, 1.9	20, 16	10, 16	0, 0	163, 31
**Vx Glu**	49, 19	0.26, 0.26	0.8, 0.4	3.0, 2.1	22, 23	13, 19	0, 0	196, 45
**P =**	0.03	0.03	0.4	0.03	0.03	0.03	0.2	0.03
**D) *Saline***							
**Rest**	44, 9	0.3, 0.0	1.0, 0.2	3.8, 0.9	35, 10	13, 12.	0, 0	202, 23
**pF**_**V**_	48, 9	0.3, 0.0	0.9, 0.2	4.0, 0.5	36, 12	14, 15	0, 0	212, 28
**P =**	0.3	0.3	0.5	0.8	0.1	0.4	0.5	0.08
**Rest**	48, 6	0.3, 0.0	0.9, 0.2	3.9, 0.7	35, 11	14, 13	0, 0	48, 6
**pF**_**L**_	48, 9	0.3, 0.0	1.0, 0.3	4.1, 0.6	35, 13	15, 13	0, 0	48, 9
**P =**	0.9	0.9	1.0	0.5	0.7	0.5	0.7	0.6

A-B) Agonists and antagonists injected into the pF_L_ (A) and pF_V_ (B). C) Glutamate injected into the pFv of vagus-intact (VI) and vagotomized (Vx) rats. D) Saline injected into the pFv or pFL. All tables display data as: median, IQR.

Disinhibition of pF_V_ neurons by unilateral injection of bicuculline increases V_T_ with a reciprocal decrease in *f* in awake rats [21]. Furthermore, pF_V_ appears to facilitate active expiration through projections to abdominal and genioglossus motoneurons, but does not itself induce active expiration [11]. We therefore expected that pF_V_ disinhibition with a cocktail of bicuculline and strychnine (B+S_pFV_) would increase V_E_, as well as alter abdominal and gengioglossus activity, but would not induce active expiration. Bilateral injections of B+S_pFV_ (n = 8) increased *f*, decreased T_I_, did not alter T_E_, and decreased V_T_ and ∫Dia_EMG_. Bilateral B+S_pFV_ in anesthetized rats did not alter V_E_ due to a compensatory decrease in V_T_ in response to an increase in *f* elicited by the antagonism of inhibitory receptors, which is the opposite response to unilateral injection of bicuculine in the same region in awake rats [21]. pF_V_ disinhibition had multiple effects on genioglossus activity, increasing inspiratory-related ∫GG_EMG_ and inducing both pre-inspiratory and post-inspiratory ∫GG_EMG_ activity (Fig 3). In 6 out of 8 experiments, B+S_pFV_ also induced high amplitude post-inspiratory Abd_EMG_ activity (Fig 3A† and 3Bii†), which while rhythmic was slow, occurring every 10 ± 1 breaths. This pattern of high amplitude post-inspiratory Abd_EMG_ activity was distinct from active expiration that occurs between every inspiration at a lower amplitude (see Fig 2 and [8, 11]. Interestingly, coincident with high amplitude post-inspiratory Abd_EMG_ bursts, there was inhibition of GG_EMG_ activity, showing co-ordination between GG_EMG_ and ABD_EMG_ during expiration (Fig 3B). In all experiments, B+S_pFV_ also induced sighs i.e., augmented breaths with high amplitude inspiratory Dia_EMG_ followed by prolonged T_E_ (Fig 3A# and 3Bii#); sighs were rhythmic but slow, occurring every 12 ± 1 breaths. The high amplitude post-inspiratory Abd_EMG_ activity was not coordinated with sighing.

To test for any nonspecific effects of pF_V_ or pF_L_ injections on breathing, we injected saline into both regions. In anesthetized rats, saline injections in the pF_L_ (n = 8) did not alter *f*, T_I_, T_E_, V_T_, ∫Dia_EMG_, GG_EMG_, ∫Abd_EMG_, or V_E_ (Fig 4). In anesthetized rats, saline injections in the pF_V_ (n = 8) did not alter *f*, T_I_, T_E_, V_T_, ∫Dia_EMG_, ∫GG_EMG_, ∫Abd_EMG_, or V_E_ (Fig 5).

### Excitation of either pF_L_ or pF_V_ affects breathing pattern (Figs 6–8, Table 2)

**Fig 6:**
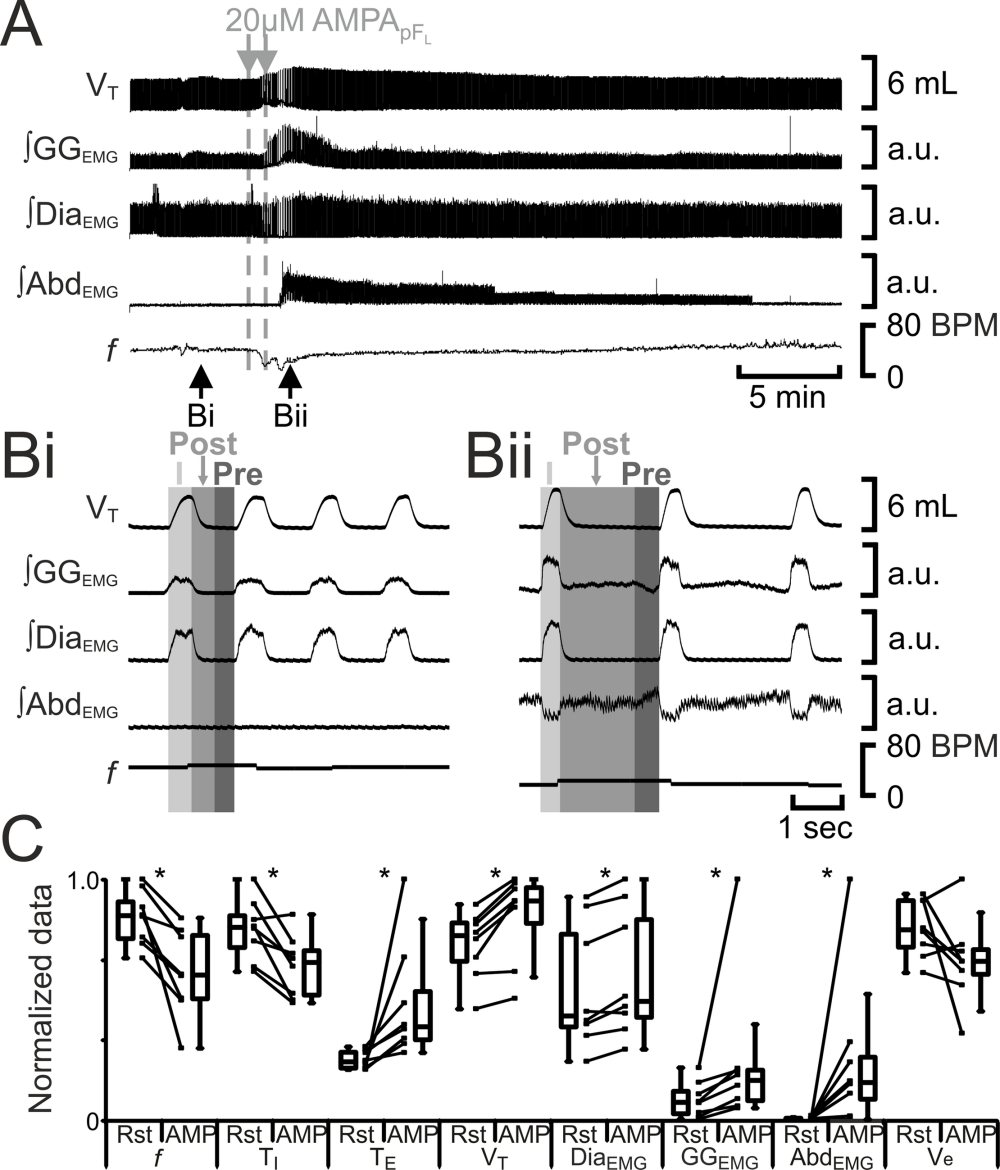
AMPA_pFL_ induces active expiration. A) Integrated traces from a single experiment. Black arrows at bottom indicate epochs in expanded traces (Bi and Bii), gray arrows at top indicate unilateral injections for AMPA_pFL_. Bi) Rest. Bii) Following AMPA_pFL_. Grey vertical boxes demark phases of each breath: inspiration (I; light gray), post-inspiration (Post: medium grey), and pre-inspiration (Pre: Dark gray). C) Comparison between ventilation at rest (Rst) and after AMPA_pFL_ injection. Lines connect data from individual experiments, box and whisker plots show combined data. Data are normalized to highest value for each parameter, i.e., *f*, T_I,_ T_E_, V_T_, GG_EMG_, Dia_EMG_, or Abd_EMG_ regardless of whether it belonged to control or AMPA_pFL_ group. *: p < 0.05. Abbreviations defined in Fig 2.

Photostimulation of pF_L_ neurons elicits active expiration [8]. We predicted that excitation of the pF_L_ with the glutamatergic agonist AMPA (AMPA_pFL_) would also elicit active expiration. Bilateral injections of AMPA_pFL_ (n = 8) decreased *f* and T_I_, and increased T_E_, V_T_, ∫Dia_EMG_, inspiratory-related ∫GG_EMG_ activity and ∫Abd_EMG_ (Fig 6), the latter a signature of active expiration, q.v., [8, 11]. Like B+S_pFL_, bilateral injections of AMPA_pFL_ did not affect V_e_, presumably due to a compensatory increase in V_T_ in response to the decrease in *f*.

**Fig 7:**
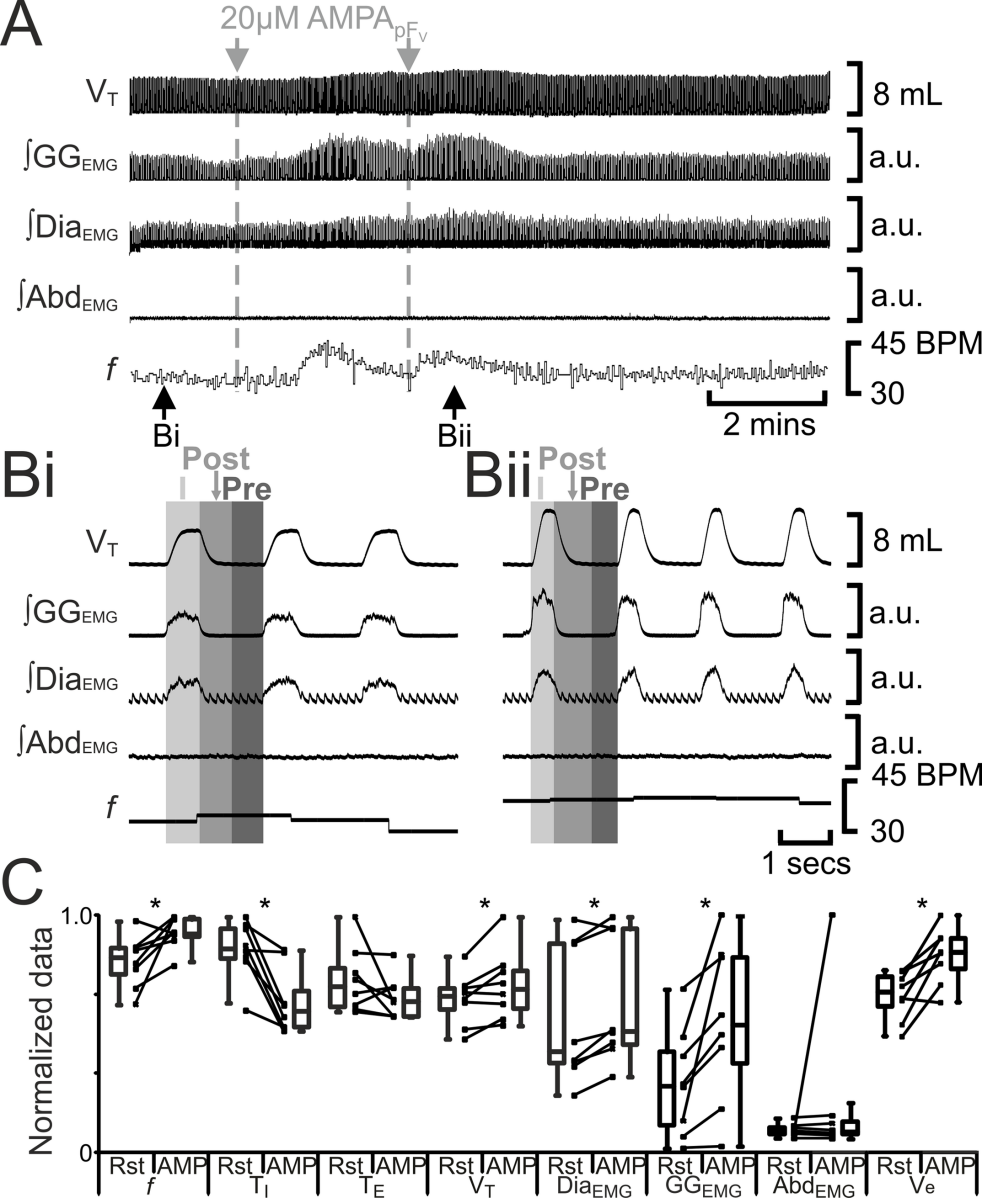
AMPA_pFV_ increases *f* and V_T_, but does not induce post-inspiratory activity in either abdominal muscles or in pre- and post-inspiratory activity gengioglossus muscles. A) Integrated traces from a single experiment. Black arrows at bottom indicate epochs in expanded traces (Bi and Bii), gray arrows at top indicate unilateral injections for AMPA_pFV_. Bi) Rest. Bii) Following AMPA_pFV_. Grey vertical boxes demark phases of each breath: inspiration (I; light gray), post-inspiration (Post: medium grey), and pre-inspiration (Pre: Dark gray). C) Comparison between ventilation at rest (Rst) and after AMPA_pFV_ injection. Lines connect data from individual experiments, box and whisker plots show combined data. Data are normalized to highest value for each parameter, i.e., *f*, T_I,_ T_E_, V_T_, GG_EMG_, Dia_EMG_, or Abd_EMG_ regardless of whether it belonged to control or AMPA_pFV_ group. *: p < 0.05. Abbreviations defined in Fig 2.

**Fig 8:**
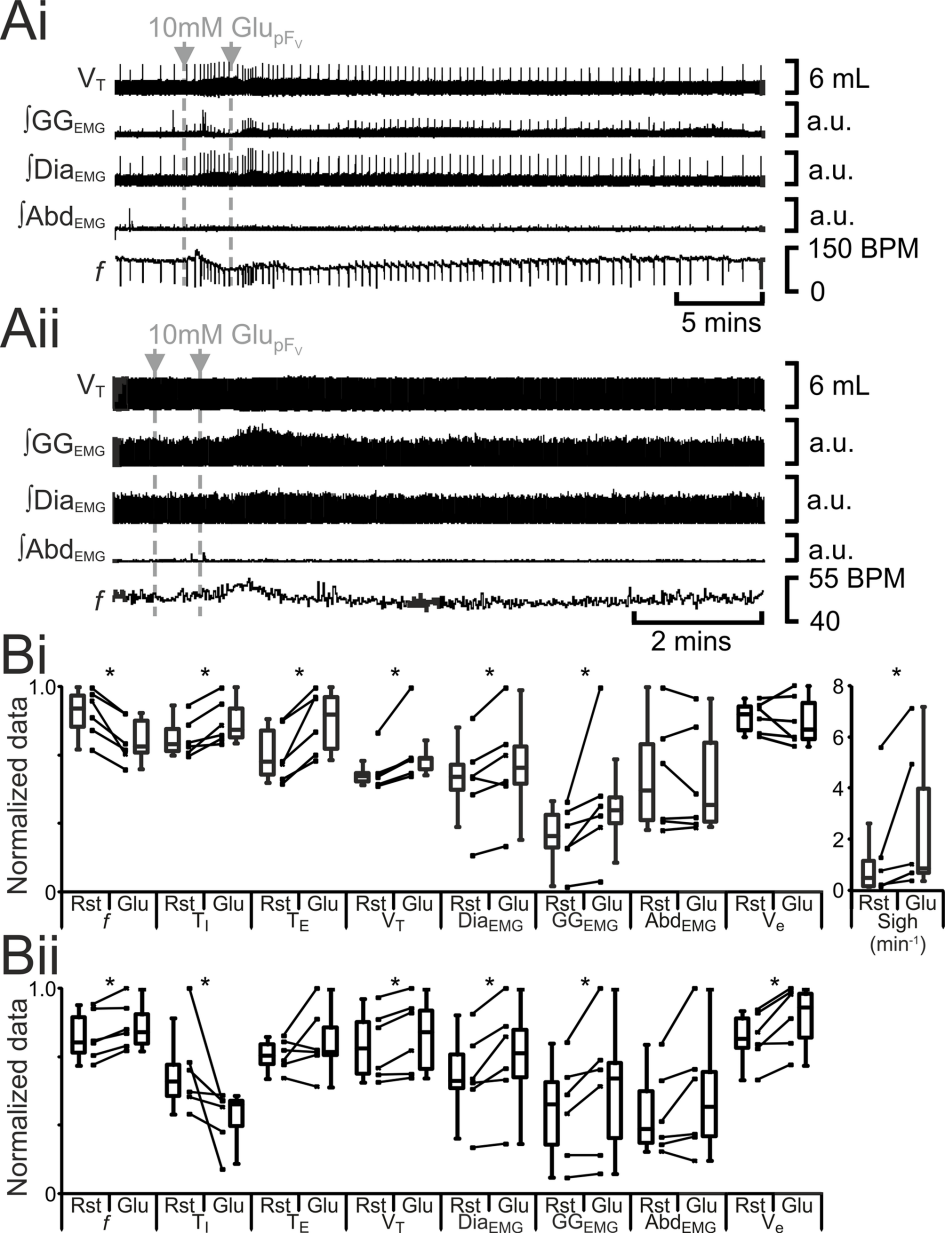
Glu_pFV_ alters, but does not induce, post-inspiratory activity in either abdominal muscles or in pre- and post-inspiratory activity in genioglossus muscles. A) Integrated traces from a single experiment, gray arrows indicate unilateral injections for Glu_pFV_. Ai) Vagus intact. Aii) Vagotomized. B) Comparison between ventilation at rest (Rst) and after Glu_pFV_. Bi) Vagus intact. Bii) Vagotomized. Lines connect data from individual experiments, box and whisker plots show combined data. Data are normalized to highest value for that parameter, i.e., *f*, T_I,_ T_E_, V_T_, GG_EMG_, Dia_EMG_, or Abd_EMG_ regardless of whether it belonged to control or Glu_pFV_ group. *: p < 0.05. Abbreviations defined in Fig 2.

Excitation of pF_V_ neurons by injection of glutamate increases phrenic nerve discharge amplitude and induces sighing in urethane anesthetized, paralyzed, artificially ventilated, vagotomized cats [22]; photostimulation of pF_V_ neurons leads to increased sighing and respiratory frequency in conscious rats [23]. We predicted that excitation of the pF_V_ with AMPA (AMPA_pFV_) would increase ventilation and sighing. Bilateral injection of AMPA_pFV_ (n = 8) increased *f*, decreased T_I_, did not alter T_E_, and increased V_T_, ∫Dia_EMG_, and inspiratory-related ∫GG_EMG_, but neither induced expiratory-modulated GG_EMG_ nor Abd_EMG_ (Fig 7). Unlike B+S_pFV_, bilateral injections of AMPA_pFV_ increased V_E_ due to increases in both V_T_ and *f*. In 5 out of 8 rats, before AMPA_pFV_ caused V_T_ to reach maximal amplitude it induced 1–2 sigh like events, but with no associated GG_EMG_ or Abd_EMG_ activity (data not shown).

The lack of induction of sighing could have been due to either the increased V_T_ in vagotomized rats, or due to the lack of activation of other glutamatergic receptors, e.g., NMDA, mGluR, etc, in addition to AMPA receptors. To explore these possibilities, in separate experiments, we injected glutamate into the pF_V_ (Glu_pFV_) of anesthetized rats before and after vagotomy. Before vagotomy (n = 8), bilateral Glu_pFV_ decreased *f*, increased T_I_, T_E_, V_T_, ∫Dia_EMG_, inspiratory-related ∫GG_EMG_ (Fig 8), and sigh rate, but neither induced expiratory-modulated GG_EMG_ nor Abd_EMG_ (Fig 8). Bilateral injections of Glu_pFV_ did not affect V_E_ due to a compensatory decrease in *f* in response to an increase in V_T_, elicited by the activation of glutamate receptors. Following vagotomy, bilateral Glu_pFV_ increased *f*, decreased T_I_, did not alter T_E_, and increased V_T_, ∫Dia_EMG_, and inspiratory-related ∫GG_EMG_, but neither induced expiratory-modulated GG_EMG_ nor Abd_EMG_ (Fig 8), similar to AMPA_pFV_ (Fig 7). Like AMPA_pFV_, bilateral injections of Glu_pFV_ increased V_E_ due to increases in both V_T_ and *f*. In 3 out of 6 vagotomized rats, before Glu_pFV_ caused V_T_ to reach maximal amplitude it induced 3–6 sigh-like events but with no associated GG_EMG_ or Abd_EMG_ (data not shown).

### Reduced excitation of pF_V_ and pF_L_ have different effects on breathing (Figs 9 and 10, Table 2)

**Fig 9:**
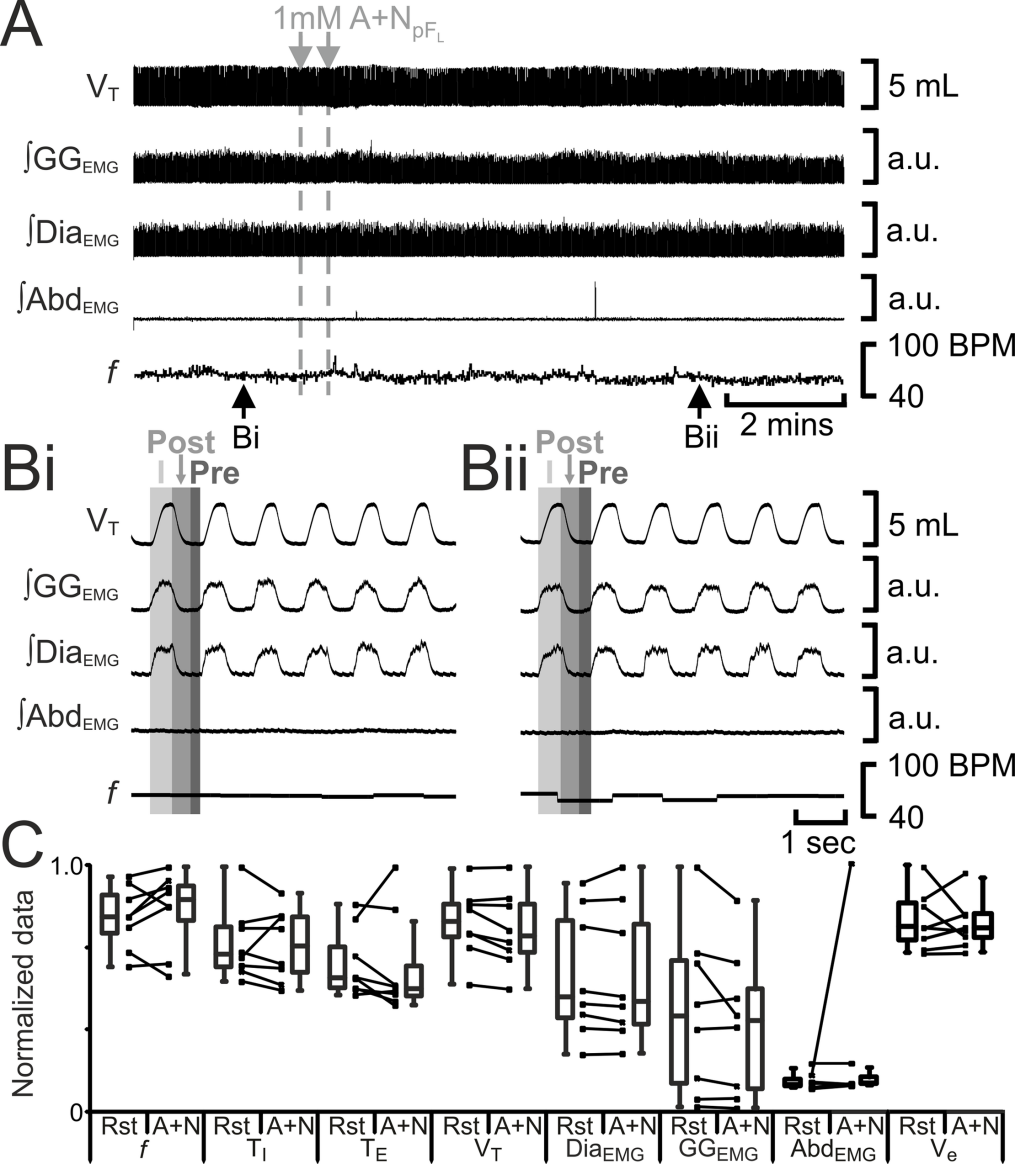
A+N_pFL_ does not affect breathing. A) Integrated traces from a single experiment. Black arrows at bottom indicate epochs in expanded traces (Bi and Bii), gray arrows at top indicate unilateral injections for A+N_pFL_. Bi) Rest. Bii) Following A+N_pFL_. Grey vertical boxes demark phases of each breath: inspiration (I; light gray), post-inspiration (Post: medium grey), and pre-inspiration (Pre: Dark gray). C) Comparison between ventilation at rest (Rst) and after A+N_pFL_ injection. Lines connect data from individual experiments, box and whisker plots show combined data. Data are normalized to highest value for each parameter, i.e., *f*, T_I,_ T_E_, V_T_, GG_EMG_, Dia_EMG_, or Abd_EMG_ regardless of whether it belonged to control or A+N_pFL_ group. Abbreviations defined in Fig 2.

Many, if not most or all, pF_L_ neurons are silent at rest [8, 24]; not surprisingly, hyperpolarizing pF_L_ neurons at rest does not affect ventilation [11]. We predicted that reduction of pF_L_ excitability with local injection of a cocktail of the glutamatergic antagonists AP-5 and NBQX (A+N_pFL_) would not affect breathing. Bilateral injections of A+N_pFL_ (n = 8) had no effect on *f*, T_I_, T_E_, V_T_, ∫Dia_EMG_, or ∫GG_EMG_; Abd_EMG_ silent at rest, remained so after A+N_pFL_ (Fig 9). Bilateral injections of A+N_pFL_ did not affect V_E_ as it neither affected V_T_ nor *f*.

**Fig 10:**
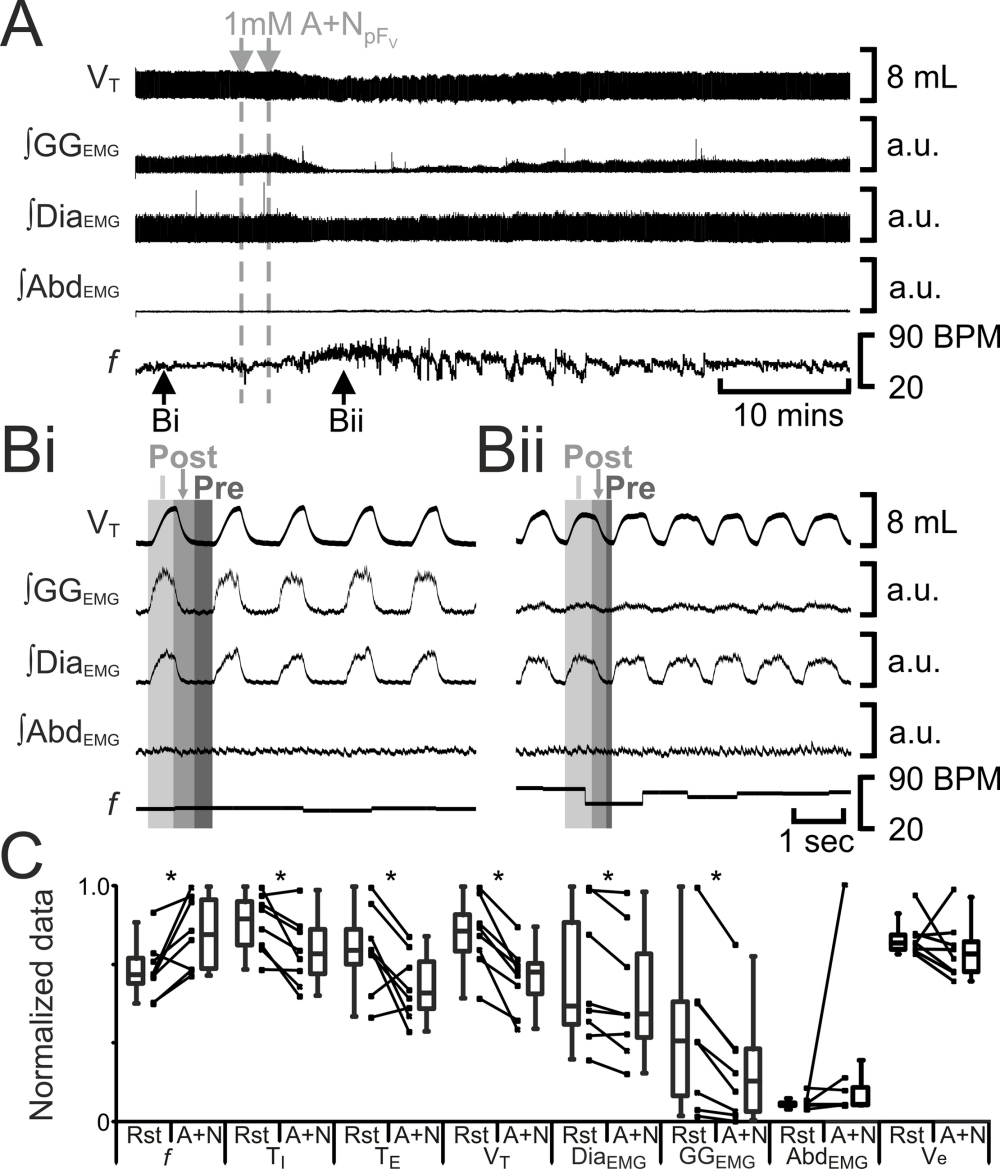
A+N_pFV_ decreases V_T_, and reduces output of inspiratory muscles. A) Integrated traces from a single experiment. Black arrows at bottom indicate epochs in expanded traces (Bi and Bii), gray arrows at top indicate unilateral injections for A+N_pFV_. Bi) Rest. Bii) Following A+N_pFV_. Grey vertical boxes demark phases of each breath: inspiration (I; light gray), post-inspiration (Post: medium grey), and pre-inspiration (Pre: Dark gray). C) Comparison between ventilation at rest (Rst) and after A+N_pFV_ injection. Lines connect data from individual experiments, box and whisker plots show combined data. Data are normalized to highest value for each parameter, i.e., *f*, T_I,_ T_E_, V_T_, GG_EMG_, Dia_EMG_, or Abd_EMG_ regardless of whether it belonged to control or A+N_pFV_ group. *: p < 0.05. Abbreviations defined in Fig 2.

By contrast, pF_V_ neurons are active at rest, providing excitatory drive for quiet breathing [25–29]; hyperpolarizing pF_V_ neurons reduces ventilation [5, 11, 13]. We predicted that reduction of pF_V_ excitability with local injection of AP-5 and NBQX (A+N_pFV_), would reduce ventilation. Bilateral A+N_pFV_ (n = 8) increased *f*, decreased T_I_ and T_E_, V_T_, ∫Dia_EMG_, and ∫GG_EMG_; Abd_EMG_, silent at rest, remained so after A+N_pFV_ (Fig 10). Bilateral injections of A+N_pFV_ did not affect V_E_ due to a compensatory increase in *f* in response to a decrease in V_T_, elicited by the activation of glutamate receptors. That no injection into the pF_V_ induced active expiration is indicative that the injectate did not spread to the adjacent pF_L_, likewise since A+N_pFL_ did not induce any changes in breathing, this indicates the injectate did not spread to the adjacent pF_V_.

## Discussion

Since the putative identification of a conditional expiratory oscillator in the rostral medulla [10, 12, 30], attention has focused on regions surrounding the facial nucleus as its location [8, 11, 15, 24, 31]. We identified two functionally separate parafacial regions: the pF_V_ and pF_L_ [11]. We propose that the pF_V_ provides a critical generic drive to breathe, driving inspiration at rest and facilitating both inspiration and expiration when chemosensory drive increases [11, 15], and that the pF_L_ is silent at rest, but once activated, drives active expiration [8, 11]. Additionally, there appears to be a third parafacial region, more dorsocaudal, containing neurons expressing gastrin releasing peptide that modulates baseline sigh rate [16]. Thus, there appear to be several distinct parafacial regions contributing to the bCPG. To further investigate the role of parafacial neurons, and the neuronal composition of parafacial regions at the ventral medullary surface, we pharmacologically altered the excitability of pF_V_ and pF_L_ neurons and measured the effects on breathing.

### Further support of the hypothesis of the pF_L_ as the source for active expiration

Antagonizing ionotropic glutamate receptors with A+N_pFL_ did not alter any respiratory parameter, i.e., no change in *f*, T_I_, T_E_, V_T_, Dia_EMG_, GG_EMG_, or Abd_EMG_, supporting our previous observation that these neurons are silent at rest, q.v., [8, 11]. In the pF_L_, excitation (with AMPA) or disinhibition (by antagonizing ionotropic GABA and glycine receptors with B+S) decreased *f* with a compensatory increase in V_T_ and inspiratory Dia_EMG_ and GG_EMG_, and onset of expiratory bursting on GG_EMG_ and Abd_EMG_, i.e., active expiration [8, 11]. Thus, these excitatory neurons have presumptive projections to neurons in the preBötzinger Complex (preBötC) or Bötzinger Complex (BötC) [32, 33] that inhibit inspiration during expiration, i.e., reciprocal inhibition, and to excitatory premotoneurons in the caudal ventral respiratory group (cVRG) that project to abdominal muscle motoneurons [34–36]. Given the delayed increase in V_T_ following the induced decrease in *f*, a direct excitatory projection from the pF_L_ to the preBötC appears unlikely, but rather suggests an indirect pathway related to controlling pCO_2_, perhaps via the pF_V_. These observations are consistent with our hypothesis that the pF_L_ is a conditional expiratory oscillator with neurons that are tonically inhibited at rest that can be turned on either by disinhibition and/or excitation.

### Multifunctional role of the pF_V_

A+N_pFV_ injected into the pF_V_ to lower its excitability, decreased V_T_ and inspiratory-related muscle activity, likely via projections to the preBötC and/or the rostral ventral respiratory group (rVRG) [37]. The associated delayed increase in *f* could again be explained as intrinsic to the slower time course of chemosensory feedback to maintain pCO_2_. As no change in phase durations or *f* were seen, it appears unlikely that this excitatory drive to inspiration was mediated by rhythmic preBӧtC neurons [38]. Rather, this observation is consistent with our hypothesis of a subpopulation of tonically active pF_V_ neurons that provides facilitative drive to phrenic and/or other inspiratory pump motoneurons to affect V_T_, but do not contribute directly to regulating *f* or inspiratory drive to genioglossal motoneurons [11]. Instead it is more likely that the pF_V_ affects V_T_ through its projections to the rVRG [39], the premotor bulbospinal relay to the phrenic nucleus for inspiratory drive [40], as this will alter V_T_ without directly altering other inspiratory parameters, i.e., *f* and GG_EMG_.

B+S_pFV_ to increase pF_V_ excitability, increased *f*, most likely through projections to the preBӧtC [38, 41], presumably to the same neurons that lead to an increase in *f* following optogenetic photostimulation of the pF_V_ [42, 43]. B+S_pFV_ also increased inspiratory-related GG_EMG_, likely through pF_V_ projections to the parahypoglossal region (pXII) [39], which appears to be the premotor relay for inspiratory drive to the XII nucleus [44]. Though B+S_pFV_ attenuated Dia_EMG_ and V_T,_ this appeared secondary to the reduction in *f* and thus was most likely due to chemosensory feedback to control pCO_2_. This further supports our hypothesis of a subpopulation of tonically suppressed pF_V_ neurons that provide facilitative drive to modulate *f*, but does not contribute directly to V_T_.

Unlike B+S_pFV_, AMPA_pFV_ potentiated V_T_ and Dia_EMG_ activity, most likely through excitation of the neurons that were attenuated by A+N_pFV_ and project to the rVRG. AMPA_pFV_ also increased *f* and inspiratory-related GG_EMG_ most likely through excitation of neurons that project to the preBӧtC and parahypoglossal region that were activated following B+S_pFV_. As B+S_pFV_ and AMPA_pFV_ each led to different patterns of breathing with neither similar to the effects of activating the pF_L_, we suggest that there are at least two relevant pF_V_ subpopulations, one expressing inhibitory receptors and one that does not, and that both of these subgroups are distinct from the pF_L_.

Similar to stimulation of pF_V_ neurons in awake behaving vagus intact rats [23] and in vagotomized urethane anesthetized cats [22], disinhibition with B+S_pFV_ elicited sighs in vagotomized rats (Fig 3Bii#), as did excitation with Glu_pFV_ in vagus-intact rats (Fig 8A). In vagotomized rats the amplitude of normal breaths is considerably larger than vagus-intact rats, with the consequence that sighs are masked. Accordingly, when V_T_ was low, i.e., in vagus-intact rats or following a reduction in amplitude caused by B+S_pFV_ in vagotomized rats, sustained increases in sigh activity could be seen. This confirms our recent study showing a cluster of neurons in the pF_V_ that release bombesin-like neuropeptides that affect sighing through the activation of cognate receptors in the preBötC [16].

Hyperpolarizing pF_V_ neurons during hypercapnia and hypoxia affects the amplitude of Abd_EMG_ and GG_EMG_, but not V_T_ or *f* [11], likely through direct projections to the cVRG [15] and parahypoglossal region [39]. Interestingly, B+S_pFV_ induced high amplitude post-inspiratory activity on both GG_EMG_ and Abd_EMG_, likely through the same projections, supporting our previous finding that the pF_V_ provides excitatory drive to expiratory premotor nuclei independent of its projections to the preBӧtC [11]. Interestingly, no perturbation of pF_V_ excitability induced active expiration, while hyperpolarization of the pF_V_ reduces active expiration during chemosensory stimulation [11, 13]. We conclude that the pF_V_ provides can modulate expiratory activity generated elsewhere, but cannot itself induce active expiration.

Interestingly, most manipulations which changed either *f* or V_T_ led to compensatory changes, presumably to regulate V_E_ to control pCO_2_ to within the normal range. For example, reducing excitation in the pF_V_ reduced activity of neurons that influence diaphragmatic (pre)motoneurons, which are constitutively active at rest. Thus, this manipulation reduced V_T_, but had no effect on *f* as the pF_V_ neurons that influence *f* were supressed at rest and therefore their activity could not be affected by A+N; this allows for other brain regions to affect preBötC rhythmogenic neurons to increase *f* to compensate for the reduction in V_T_. Only one manipulation, glutamatergic activation of the pF_V_ (with either AMPA or Glu) changed V_E_. We believe that this is because glutamatergic activation of the pF_V_ RTN leads to activation of the tonically supressed neurons that activate preBötC rhythmogenic neurons; furthermore this manipulation also excites the neurons that are active at rest that influence diaphragmatic (pre)motoneurons, consequently altering both *f* and V_T_ simultaneously.

## Summary

**Fig 11:**
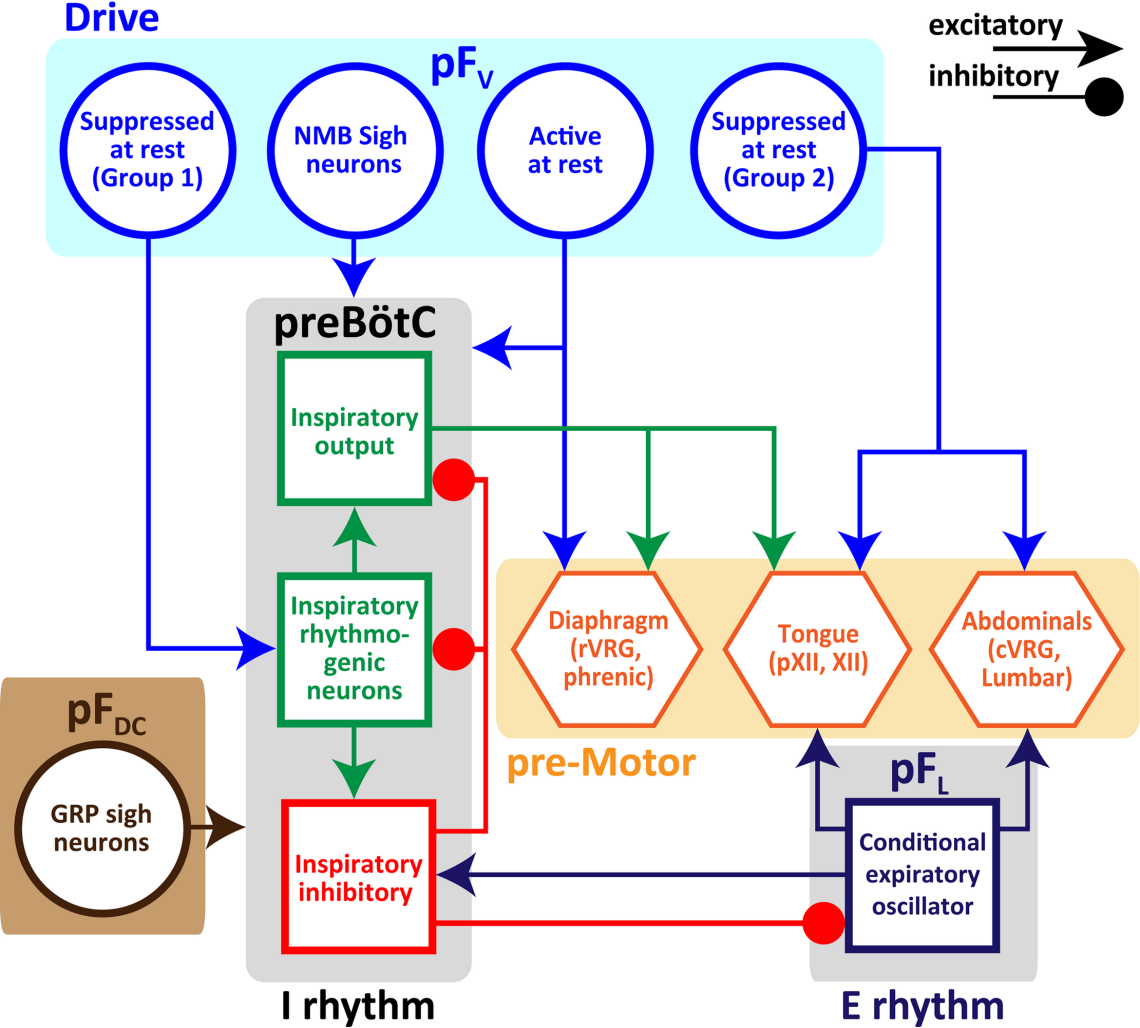
Schematic of minimal bCPG, which consists of 4 essential components. 1) preBötzinger Complex (preBötC) drives inspiration by exciting inspiratory premotor neuronal populations projecting to inspiratory muscles, e.g., diaphragm and tongue, and inhibits pF_L_; 2) parafacial Dorsocaudal (pF_DC_) contains GRP positive neurons contributing to basal sigh rhythm. 3) pF_L_ drives active expiration by exciting expiratory premotor neuronal populations projecting to expiratory muscles, e.g., abdominals and tongue, and excites neurons that inhibit preBötC, either in preBötC or in BötC (not shown); 4) pF_V_ contains neurons and glia that contribute to CO_2_/pH regulation and integrates sensory afferents affecting breathing, including basal sigh rate, via excitatory connections to preBötC and breathing premotor and motor neurons. pF_V_ contains 4 subpopulations: i) tonically active neurons that modulate V_T_ and diaphragm bursting at rest; ii) tonically suppressed neurons that modulate *f*; iii) NMB positive neurons that affect basal sigh rate, and; iv) tonically suppressed neurons that provide rhythmic drive to abdominal and genioglossus expiratory motor pools producing active expiration.

We propose that there are at least 6 subpopulations of parafacial neurons (Fig 11). The pF_L_ is a conditional expiratory oscillator, with a functionally homogeneous population of neurons that drive active expiration (Fig 11). By contrast, the pF_V_ provides a critical generic facilitatory drive to breathe, and consists of at least 4 functionally distinct subpopulations of neurons: i) a tonically active subpopulation that drives V_T_ via the diaphragm; ii) one subpopulation of tonically suppressed neurons that modulate *f*; and; iii) a second subpopulation of tonically suppressed neurons that provide drive to abdominal and genioglossus expiratory motor pools, iv) a subpopulation of bombesin-peptide, i.e., NMB, neurons of the hypothesized peptidergic sigh circuit [16]. In addition, there is a 6^th^ subpopulation bombesin-peptide, i.e., GRP, neurons in the dorsocaudal parafacial (pF_DC_) that also can modulate basal sigh rate [16].
